# Does the clot burden as assessed by the Mean Bilateral Proximal Extension of the Clot score reflect mortality and adverse outcome after pulmonary embolism?

**DOI:** 10.1177/20584601231187094

**Published:** 2023-06-29

**Authors:** Jostein Gleditsch, Øyvind Jervan, Frederikus Klok, René Holst, Einar Hopp, Mazdak Tavoly, Waleed Ghanima

**Affiliations:** 1 Department of Radiology, 60517 Østfold Hospital, Kalnes, Norway; 2 Institute of Clinical Medicine, 60504 University of Oslo Faculty of Medicine, Oslo, Norway; 3 Department of Cardiology, 60517 Østfold Hospital, Kalnes, Norway; 4 Department of Medicine – Thrombosis and Hemostasis, 4501 Leiden University Medical Center, Leiden, The Netherlands; 5 Oslo Centre for Biostatistics and Epidemiology, University of Oslo and Oslo University Hospital, Oslo, Norway; 6 Division of Radiology and Nuclear Medicine, 155272 Oslo University Hospital, Oslo, Norway; 7 Department of Medicine, 56749 Sahlgrenska University Hospital, Gothenburg, Sweden; 8 Internal Medicine Clinic, 60517 Østfold Hospital, Kalnes, Norway; 9 Department of Hematology, Oslo University Hospital and Institute of Clinical Medicine, 6305 University of Oslo, Oslo, Norway

**Keywords:** Computed tomography angiography, risk assessment, pulmonary artery/diagnostic imaging, pulmonary embolism/mortality, retrospective studies

## Abstract

**Background:**

Rapid diagnosis and risk stratification are important to reduce the risk of adverse clinical events and mortality in acute pulmonary embolism (PE). Although clot burden has not been consistently shown to correlate with disease outcomes, proximally located PE is generally perceived as more severe.

**Purpose:**

To explore the ability of the Mean Bilateral Proximal Extension of the Clot (MBPEC) score to predict mortality and adverse outcome.

**Methods:**

This was a single center retrospective cohort study. 1743 patients with computed tomography pulmonary arteriography (CTPA) verified PE diagnosed between 2005 and 2020 were included. Patients with active malignancy were excluded. The PE clot burden was assessed with MBPEC score: The most proximal extension of PE was scored in each lung from 1 = sub-segmental to 4 = central. The MBPEC score is the score from each lung divided by two and rounded up to nearest integer.

**Results:**

We found inconsistent associations between higher and lower MBPEC scores versus mortality. The all-cause 30-day mortality of 3.9% (95% CI: 3.0–4.9). The PE-related mortality was 2.4% (95% CI: 1.7–3.3). Patients with MBPEC score 1 had higher all-cause mortality compared to patients with MBPEC score 4: Crude Hazard Ratio (cHR) was 2.02 (95% CI: 1.09–3.72). PE-related mortality was lower in patients with MBPEC score 3 compared to score 4: cHR 0.22 (95% CI: 0.05–0.93). Patients with MBPEC score 4 did more often receive systemic thrombolysis compared to patients with MBPEC score 1–3: 3.2% vs. 0.6% (*p* < .001). Patients with MBPEC score 4 where more often admitted to the intensive care unit: 13% vs. 4.7% (*p* < .001).

**Conclusion:**

We found no consistent association between the MBPEC score and mortality. Our results therefore indicate that peripheral PE does not necessarily entail a lower morality risk than proximal PE.

## Introduction

Acute pulmonary embolism (PE) is a potentially fatal condition with a 30-day mortality rate ranging from 1–3% in low-risk patients and up to 15% in high-risk patients.^1–3^ Rapid diagnosis and evidence-based risk stratification are essential to reduce the risk of adverse clinical events and mortality.^4,5^

Computed tomography pulmonary angiography (CTPA) allows rapid and accurate diagnosis of PE and is universally embraced as the current gold standard diagnostic tool for PE.^6,7^ Proper risk stratification following the diagnosis of PE is essential to determine the appropriate management.^8^ Patients with the highest risk of adverse outcomes, that is, those who present with obstructive shock, require immediate reperfusion treatment and admission to the medical intensive care unit, in contrast to low-risk patients who can be managed on an outpatient basis. Current risk stratification guidelines consist of hemodynamic evaluation including the Pulmonary Embolism Severity Index (PESI) score,^9^ followed by the evaluation of right heart dysfunction and laboratory biomarkers of myocardial injury.^8^

Right to left ventricle (RV/LV) ratio, as assessed by CTPA or echocardiography, has been established as a prognostic marker for adverse outcomes following PE^10–12^ and is part of current risk stratification algorithms in acute PE. In contrast, clot burden (proximal extension of the clot or degree of arterial obstruction) per se has not been consistently shown to correlate to patient outcomes.^13^ However, from a clinical perspective, clot burden is often used as a measure for PE severity and a guide for management. Centrally located PE is generally perceived as more severe, despite lacking evidence.^11,14–17^ Several methods for clot burden assessment are available, such as the Mastora^18^ and Qanadli^19^ scores. These scores are based on the number of affected pulmonary segments and the degree of pulmonary artery obstruction. A number of small studies have shown an association between clot burden and mortality,^20,21^ whereas others have failed to demonstrate such associations.^22,23^ Due to complexity and lack of clinical consequences, neither Mastora nor Qanadli scores have been implemented in routine clinical practice. Therefore, a radiological score based on the Mean Bilateral Proximal Extension of the Clot (MBPEC) was proposed.^24^ The MBPEC score is based solely on CTPA assessment and the scoring can be completed quickly in addition to the ordinary CTPA interpretation. A strong association between the MBPEC score and the Qanadli score has previously been demonstrated (r = 0.9).^24^ In a more recent study, the MBPEC score was shown to correlate with PESI score, RV/LV ratio and cardiac biomarkers beside several other PE related clinical markers.^25^ However, the ability of the MBPEC score to predict mortality and other adverse outcomes has not been studied previously.

Accordingly, the primary aim of the present study was to determine the prognostic value of the MBPEC score in predicting 30-day mortality in PE patients. Secondary aims were to determine the prognostic value of the MBPEC score in predicting other adverse clinical outcomes, including the need for cardiopulmonary resuscitation, mechanical ventilation support, systemic thrombolysis, and admission to the intensive care unit as well as to determine the prognostic value of the unilateral most proximal extension of the clot irrespective of which side. The latter is frequently used parameter by clinician and often influence the perception of the severity.

## Material and methods

### Study design

This was a single center retrospective cohort study. The study was approved by the Regional Committee for Medical and Health Research Ethics in Norway (REK 2017/1329). All participants provided informed written consent unless they were deceased, where consent was waived by REK.

Patients were identified through the Østfold Thrombosis Registry (TROLL).^26^ The TROLL registry includes all patients who have been diagnosed with and/or treated for venous thromboembolism (VTE) at Østfold Hospital, Norway, from January 2005 and onwards (NSD approval no 28435/5/LMR/LR).

Patients who met the following criteria were included in the present study: (1) Registered in TROLL with the diagnosis of PE (both symptomatic and incidental) during the period 2005 to 2020 and (2) PE diagnosis confirmed by CTPA. Patients incidentally diagnosed with PE after contrast-enhanced computed tomography (CT) were also included when image quality was sufficient to confirm PE and calculate MBPEC score. Only the first PE-episode registered in TROLL was included in the study.

Exclusion criteria included (1) insufficient CTPA image quality to confirm the PE diagnosis or to perform MBPEC assessment, (2) unavailable clinical data (mainly due to transferal from another hospital), and (3) active malignancy. Active malignancy was defined as cancer within the past 6 months, ongoing active chemotherapy, or recurrent or metastatic disease. Patients with squamous skin cancer and basal cell carcinoma were not excluded.

### Computed tomography pulmonary arteriography

All included patients were subjected to contrast-enhanced CT or CTPA as an initial diagnostic evaluation according to the routine imaging protocol used at the time of diagnosis. Low-osmolar or iso-osmolar contrast material was injected through the cubital vein by a power injector according to the current clinical routine. CT-scans were obtained with 4-slice, 40-slice, 64-slice or 128-slice scanners (Phillips MX8000, Phillips Brilliance 40, Phillips Brilliance 64, Philips Ingenuity 128; Eindhoven, the Netherlands or Toshiba Aquilion ONE; Tochigi, Japan). Images were acquired in the caudocranial direction and reconstructed to 3 mm slices in the transversal plane (from 2006, with the addition of reconstructions in the sagittal and coronal planes).

For the current study, all CTPA examinations were reassessed by an experienced radiologist to confirm the PE diagnosis, and to determine the MBPEC score. The following main diagnostic criteria for PE were used in the CTPA reassessment:^27^ A complete or partial filling defect causing failure to enhance the entire pulmonary artery lumen. The radiologist responsible for CTPA reassessments was blinded to the outcome, medical records and laboratory results, but had access to the original clinical CTPA request forms and reports.

### CTPA analyses

Mean Bilateral Proximal Extension of the Clot was used to assess the clot burden.^24^ For each lung, the most proximal extension of the embolus was assigned a number as follows: (1) for sub-segmental PE, (2) for segmental PE, (3) for lobar PE, and (4) for central PE, that is, involving the pulmonary trunk or main pulmonary arteries. The final MBPEC score was the mean of the category values from both lungs rounded up to the closest integer. For example, a sub-segmental embolism on the left side and a central embolism on the right side corresponded to (1 + 4)/2 = 2.5, and when rounded up the final MBPEC score of 3. In addition to MBPEC, the most proximal extension of PE irrespective of side was also recorded, that is, sub-segmental, segmental, lobar, or central PE.

### Descriptive parameters

The medical records at the time of PE diagnosis were reviewed by two residents blinded to MBPEC scores. The following descriptive clinical parameters and laboratory results from the time of diagnosis were acquired: VTE history, previous cancer (patients with active malignancy were excluded), and comorbidities. In 2010–2011, there was a shift from in treatment from Warfarin to direct oral anticoagulants. In 2015 a newly built hospital was brought into use, leading to major changes in workflow and available radiology equipment. Assuming these changes impacting the outcome of the study, the year of PE diagnosis was categorized into three time periods: 2005–2010, 2011–2015, and 2016–2020. Pulmonary Embolism Severity Index score was retrospectively calculated based on information from the medical records and categorized into PESI class I-II (low-risk) and class III–V (high risk) according to current guidelines.^12^ Comorbidities were categorized according to the Charlson comorbidity index.^28^ Age at diagnosis and survival time was calculated from the date of birth and date of death, which were obtained from the Norwegian national population registry.

### Study outcomes

PE-related death was defined according to the International Society on Thrombosis and Haemostasis (ISTH) guidelines,^29^ that is, autopsy-confirmed PE in the absence of another more likely cause of death, objectively confirmed PE before death in the absence of another more likely cause of death, or PE not objectively confirmed but assessed as the most likely main cause of death. Medical records of all patients who died within 30 days of the acute PE event were independently reviewed by two cardiologists to assess whether the cause of death was related to PE. In case of disagreement, the final decision was reached by consensus.

The following adverse clinical outcomes were recorded from the patients’ medical records: need for cardiopulmonary resuscitation (CPR), mechanical ventilation support, thrombolysis, or admission to the intensive care unit. Cardiopulmonary resuscitation was defined as the need for chest compressions and/or mechanical ventilation. Mechanical ventilation was defined as the need for respiratory assistance with endotracheal intubation or non-invasive ventilation. Systemic thrombolysis was defined as the intravenous administration of a thrombolytic agent.

### Statistical analysis

Continuous variables were reported as medians with corresponding interquartile ranges (IQRs) and were compared with Wilcoxon rank-sum test. Proportions were compared with chi-squared test. Thirty-day mortality was reported as case fatality rates with 95% confidence intervals based on the Poisson distribution. The effects on mortality of the MBPEC score and the most proximal extension of the PE, was presented with crude Hazard Ratios (HR) and Hazard Ratios adjusted for age (grouped by < 60 years, 60–70 years, 70–80 years or >80 years), sex, comorbidities (Charlson comorbidity index score grouped by 0, 1, 2, or ≥3), and year of diagnosis (grouped by 2005–2010, 2011–2015, or 2016–2020).

All-cause and PE-related 30-day mortality stratified by MBPEC score and the most proximal extension of the PE, respectively, are presented with Nelson–Aalen cumulative hazard plots. A sensitivity analysis was performed in patients without comorbidities (Charlson comorbidity index score of 0), and in patients with PESI class I–II and PESI class III–V, respectively.

A *p*-value less than .05 was considered statically significant. All tests were two-sided. Missing values were not imputed. Corrections for multiple comparisons were not performed. All statistical analyses were performed using Stata version 17.0 (StataCorp LLC, College Station, TX, USA).

## Results

**Figure 1.**
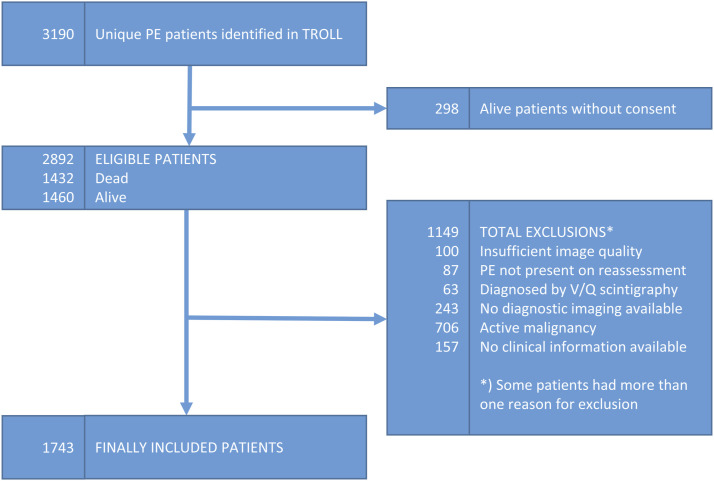
Inclusion and exclusion flowchart.

3190 PE patients were identified through the TROLL registry and assessed for eligibility (Figure 1). After excluding patients with inadequate CT image quality, unavailable clinical information or active malignancy, 1743 patients remained and were included in the final study cohort.

Demographic parameters are displayed in Table 1. Median age was 69 years (IQR: 57–79 years) and 908 (52%) were men. Chronic obstructive pulmonary disease (COPD), myocardial infarction, diabetes, heart failure, and connective tissue disease were the most frequent comorbidities. Fifty-eight percent of the patients had no comorbidities.

**Table 1.** Demographic parameters, year of PE diagnosis, comorbidities as assessed with Charlson comorbidity index and PESI score. Medians with IQR or number of patients with proportions. All patients and patients that died within 30 days irrespective of cause.

	All patients	Dead patients	*p*
*N*	1743	68	

Age, median (IQR)	69 (57–79)	79 (70–86)	<0.001
<60 years	503 (30)	5 (7)	<0.001
60–70 years	397 (24)	12 (18)	
70–80 years	408 (24)	18 (26)	
>80 years	367 (22)	33 (49)	

Sex (men)	908 (52)	31 (46)	ns

Year of PE diagnosis			
2005–2010	350 (20)	13 (19)	0.002
2011–2015	618 (35)	37 (54)	
2016–2020	775 (44)	18 (26)	

Previous VTE			
Previous DVT	125 (7)	4 (6)	ns
Previous PE	55 (3)	3 (4)	ns

Previous cancer	171 (10)	8 (12)	ns

Comorbidities^a^			
COPD	166 (10)	10 (15)	ns
Heart failure	121 (7)	19 (28)	<0.001
Heart infarction	166 (10)	12 (18)	0.020
Peripheral artery disease	56 (3)	1 (1)	ns
Brain infraction	100 (6)	7 (10)	ns
Dementia	63 (4)	10 (15)	<0.001
Diabetes	148 (8)	7 (10)	ns
Renal insufficiency^b^	68 (4)	13 (19)	<0.001
Connective tissue disease^c^	122 (7)	6 (9)	ns
Gastric ulcer	53 (3)	1 (1)	ns

Charlson comorbidity index			
Score 0	1016 (58)	16 (24)	<0.001
Score 1	354 (20)	17 (25)	
Score 2	200 (11)	16 (24)	
Score ≥3	173 (10)	19 (28)	

PESI class			
I–II	1034 (59)	16 (24)	<0.001
III–IV	709 (41)	52 (76)	

IQR: interquartile range; VTE: venous thromboembolism; DVT: deep venous thrombosis; PE: pulmonary embolism; PESI: pulmonary embolism severity index; COPD: chronic obstructive pulmonary disease.

^a^Comorbidities from Charlson comorbidity index with more than 50 registered patients.

^b^Estimated glomerular filtration rate <45 mL/min/1.73 m^2^.

^c^Including fibromyalgia.

Sixty-eight deaths occurred within 30 days from PE diagnosis, of which 42 deaths were considered PE-related. The overall all-cause 30-day case fatality rate was 3.9% (95% CI: 3.0–4.9), whereas the PE-related case fatality rate was 2.4% (95% CI: 1.7–3.3).

### Effects of clot burden on mortality

**Figure 2.**
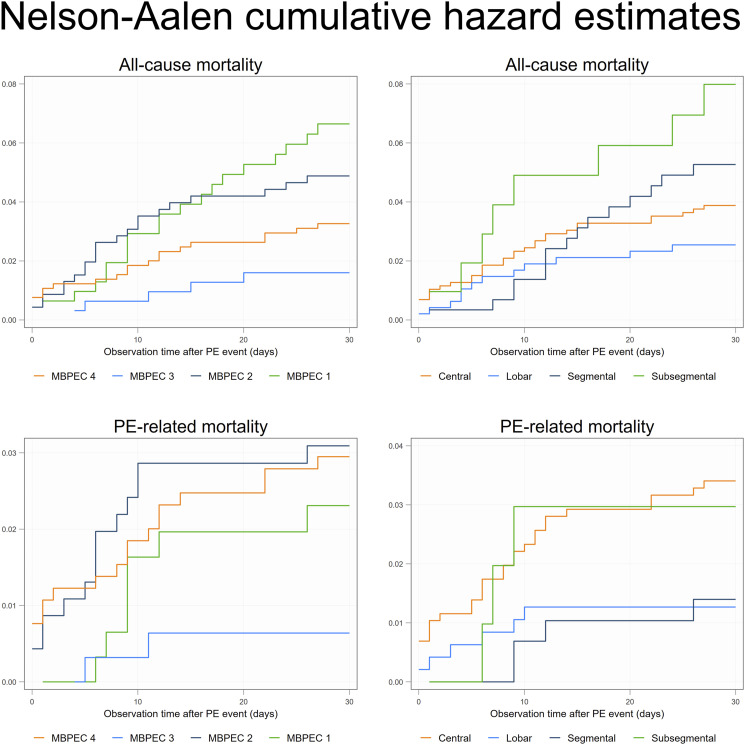
Nelson Aalen curves showing the cumulative hazard of all-cause mortality and PE-related mortality stratified by mean bilateral proximal extension of the clot (MBPEC) and by most proximal extension of the clot (irrespective side).

Associations between all-cause 30-day mortality and PE-related mortality stratified by the MBPEC score are presented in Table 2 and as Nelson-Aalen cumulative hazard plots in Figure 2. Patients with MBPEC score 1 had higher all-cause mortality compared to patients with MBPEC score 4: Crude HR 2.02 (95% CI: 1.09–3.72). PE-related mortality was lower in patients with MBPEC score 3 compared to MBPEC score 4: Crude HR 0.22 (95% CI: 0.05–0.93). However, this association was not confirmed after adjustment for age, sex, comorbidities and year of PE. Mean Bilateral Proximal Extension of the Clot score was neither associated with all-cause nor with PE-related mortality in the sensitivity analysis of patients with Charlson comorbidity index score of 0, or in patients with PESI class I–II (Table 3). Mean Bilateral Proximal Extension of the Clot score 1 was associated with higher mortality compared to MBPEC score 4 in patients with PESI class III–V: Crude HR 2.33 (95% CI: 1.17–4.68).

**Table 2.** All-cause 30-day mortality and PE-related 30-day mortality versus extension of the clot. Absolute numbers of deaths and case fatality rates. Crude and adjusted hazard ratios with 95% confidence intervals.

	All patients	All-cause mortality			PE-related mortality		
		Dead patients (CFR)	Crude HR (95% CI)	Adjusted HR^a^ (95% CI)	Dead patients (CFR)	Crude HR (95% CI)	Adjusted HR^a^ (95% CI)
All patients	1743	68 (3.9)			42 (2.4)		
MBPEC							
Score 4	655 (38)	21 (3.2)	Ref	Ref	19 (2.9)	Ref	Ref
Score 3	315 (18)	5 (1.6)	ns	ns	2 (0.6)	0.22 (0.05–0.93)^*^	ns
Score 2	462 (27)	22 (4.8)	ns	ns	14 (3.0)	ns	ns
Score 1	311 (18)	20 (6.4)	2.02 (1.09–3.72)^*^	1.96 (1.05–3.65)^*^	7 (2.3)	ns	ns
Most proximal extension of PE							
Central PE	869 (50)	33 (3.8)	Ref	Ref	29 (3.3)	Ref	Ref
Lobar PE	478 (27)	12 (2.5)	ns	ns	6 (1.3)	0.37 (0.16–0.90)^*^	ns
Segmental PE	292 (17)	15 (5.1)	ns	ns	4 (1.4)	ns	ns
Sub-segmental PE	104 (6)	8 (7.7)	ns	2.78 (1.25–6.14)^*^	3 (2.9)	ns	ns

MBPEC: mean bilateral proximal extension of the clot; PE: pulmonary embolism; CI: confidence interval; HR: hazard ratio; CFR: case fatality rate; ref: reference; ns: not significant.

^*^p < .05.

^a^Adjusted for age, sex, number of comorbidities, and year of PE diagnosis.

**Table 3.** Sensitivity analyses. All-cause 30-day mortality and PE-related 30-day mortality in patients with Charlson comorbidity index score of 0, PESI class I–II and PESI class III–V separately. Absolute numbers of deaths and case fatality rates. Crude and adjusted hazard ratios with 95% confidence intervals.

	Total (%)	All-cause mortality			PE-related mortality		
		Dead patients (CFR)	Crude HR (95% CI)	Adjusted HR^a^ (95% CI)	Dead patients (CFR)	Crude HR (95% CI)	Adjusted HR^a^ (95% CI)
Patients with CCI score of 0	1016	16 (1.6)			10 (1.0)		
MBPEC							
Score 4	398 (39)	6 (1.5)	Ref	Ref	6 (1.5)	Ref	Ref
Score 3	209 (21)	2 (1.0)	ns	ns	1 (0.5)	ns	ns
Score 2	248 (24)	4 (1.6)	ns	ns	2 (0.8)	ns	ns
Score 1	161 (16)	4 (2.5)	ns	ns	1 (0.6)	ns	ns

Most proximal extension of PE							
Central PE	553 (51)	10 (1.8)	Ref	Ref	9 (1.8)	Ref	Ref
Lobar PE	308 (28)	2 (0.6)	ns	ns	0	—	—
Segmental PE	170 (16)	4 (2.4)	ns	ns	0	—	—
Sub-segmental PE	60 (6)	1 (1.7)	ns	ns	1 (1.7)	ns	ns

Patients with PESI class I–II	1034	16 (1.6)			12 (1.2)		
MBPEC							
Score 4	374 (36)	6 (1.6)	Ref	Ref	6 (1.6)	Ref	Ref
Score 3	217 (21)	1 (0.5)	ns	ns	0	—	—
Score 2	271 (26)	6 (2.2)	ns	ns	4 (1.5)	ns	ns
Score 1	172 (17)	3 (1.7)	ns	ns	2 (1.2)	ns	ns
Most proximal extension of PE							
Central PE	490 (47)	8 (1.6)	Ref	Ref	7 (1.4)	Ref	Ref
Lobar PE	317 (31)	5 (1.6)	ns	ns	3 (1.0)	ns	ns
Segmental PE	163 (16)	1 (0.6)	ns	ns	0	—	—
Sub-segmental PE	64 (6)	2 (3.1)	ns	ns	2 (3.1)	ns	ns

Patients with PESI class III–V	709	52 (7.3)			30 (4.2)		
MBPEC							
Score 4	281 (40)	15 (5.3)	Ref	Ref	13 (4.6)	Ref	Ref
Score 3	98 (14)	4 (4.1)	ns	ns	2 (2.0)	ns	ns
Score 2	191 (27)	16 (8.4)	ns	ns	10 (5.2)	ns	ns
Score 1	139 (20)	17 (12.2)	2.33 (1.17–4.68)^*^	2.38 (1.17–4.85)^*^	5 (3.6)	ns	ns
Most proximal extension of PE							
Central PE	379 (53)	25 (6.6)	Ref	Ref	22 (5.8)	Ref	Ref
Lobar PE	161 (23)	7 (4.4)	ns	ns	3 (1.9)	ns	ns
Segmental PE	129 (18)	14 (10.9)	ns	ns	4 (3.1)	ns	ns
Sub-segmental PE	40 (6)	6 (15)	ns	3.07 (1.22–7.70)^*^	1 (2.5)	ns	ns

MBPEC: mean bilateral proximal extension of the clot; PE: pulmonary embolism; CI: confidence interval; HR: hazard ratio; CFR: case fatality rate; CCI: Charlson comorbidity index; ref: reference; ns: not significant.

^*^p < .05.

^a^Adjusted for age, sex, number of comorbidities, and year of PE diagnosis.

Similar mortality estimates were found after classification by the most proximal extension of the clot (irrespective of which side). No associations were detected, except an adjusted HR of 3.07 (95% CI: 1.22–7.70) for all-cause mortality in sub-segmental PE compared to central PE in patients with PESI class III–V.

### Effects of clot burden on adverse clinical outcomes

Adverse clinical outcomes are presented in Table 4. Eighteen (1.0%) patients received CPR, 19 (1.1%) mechanical ventilation support, and 27 (1.6%) systemic thrombolysis. 133 (7.8%) patients were admitted to the intensive care unit.

**Table 4.** Adverse clinical outcomes.

	Total (%)	MBPEC score 1–3	MBPEC score 4	*p*
**All patients**	1743	1088	655	
Cardiopulmonary resuscitation	18 (1.0)	6 (0.6)	12 (1.8)	0.01
Mechanical ventilation support	19 (1.1)	9 (0.8)	10 (1.5)	ns
Systemic thrombolysis	27 (1.6)	6 (0.6)	21 (3.2)	<0.001
Admitted to the intensive care unit	133 (7.8)	50 (4.7)	83 (13.0)	<0.001

**Patients with CCI score of 0**	1016	618	398	
Cardiopulmonary resuscitation	11 (1.1)	4 (0.7)	7 (1.8)	ns
Mechanical ventilation support	7 (0.7)	3 (0.5)	4 (1.1)	ns
Systemic thrombolysis	14 (1.4)	2 (0.3)	12 (3.0)	<0.001
Admitted to the intensive care unit	58 (5.8)	19 (3.1)	39 (10.0)	<0.001

**Patients with PESI class I-II**	1034	660	374	
Cardiopulmonary resuscitation	5 (0.5)	1 (0.2)	4 (1.1)	ns
Mechanical ventilation support	3 (0.3)	1 (0.2)	2 (0.5)	ns
Systemic thrombolysis	6 (0.6)	1 (0.2)	5 (1.3)	0.02
Admitted to the intensive care unit	47 (4.6)	14 (2.2)	33 (9.0)	<0.001

**Patients with PESI class III–V**	689	416	273	
Cardiopulmonary resuscitation	13 (1.8)	5 (1.2)	8 (2.9)	ns
Mechanical ventilation support	16 (2.3)	8 (1.9)	8 (1.9)	ns
Systemic thrombolysis	86 (12.5)	36 (8.7)	50 (18.3)	0.001
Admitted to the intensive care unit	86 (12.5)	36 (8.7)	50 (18.3)	<0.001

MBPEC: mean bilateral proximal extension of the clot; CCI: Charlson comorbidity index; ns: not significant.

We found a higher frequency of adverse clinical outcome in patients with MBPEC score 4 compared to patients with MBPEC score 1–3. Patients with MBPEC score 4 more frequently received systemic thrombolysis compared to patients with MBPEC score 1–3 (*p* < .001). Furthermore, patients with MBPEC score 4 were more frequently admitted to the intensive care unit (*p* < .001). Restricting the analysis to patients with Charlson comorbidity index score of 0, PESI class I-II or PESI class III–V revealed similar results. Patients with MBPEC score 4 more frequently received CPR compared to patients with MBPEC score 1–3 (*p* < .01). However, this association was not retained in the sensitivity analyses.

## Discussion

In the present study, we explored the association between clot burden, as assessed by the MBPEC score and mortality. We found no clinically significant association between mortality and the MBPEC score or the most proximal extension of PE. Furthermore, we found that patients with MBPEC score 4 more frequently received systemic thrombolysis, and were more frequently admitted to the intensive care unit.

In our cohort, the 30-day all-cause mortality was 3.9% and the PE-related mortality was 2.4%, which is lower than those reported in previous studies. In a study of 906 consecutive patients with PE, all-cause and PE-related 30-day death rates were reported at 7.2% (95% CI: 5.5–8.8%) and 4.1% (95% CI: 2.8–5.4%), respectively.^30^ In an epidemiological study from Australia including data from 2002 to 2018 reported a 30-day all-cause mortality rate of 5.6%.^31^ One explanation for the lower mortality rates in our cohort may be the inclusion of incidental PE. As for most studies, we cannot exclude that the low mortality in our study might be influenced by the lack of inclusion of patients with severe PE who died prior to hospital admission, and therefore were not subjected to CT and as such were not registered in the TROLL registry. However, the present study includes nearly all patients diagnosed with PE in our region. Thus, we consider our population to be representative of a true PE population.

Our results indicate a modestly higher all-cause 30-day mortality in MBPEC score 1 compared to MBPEC score 4. However, we consider it difficult to find a pathophysiological explanation for this association. Therefore, we cannot exclude that this finding may be explained by unmeasured confounding. For example, some patients might have incidentally been diagnosed with peripheral PE discovered during CT examinations performed for other reasons. Due to this, the higher mortality in MBPEC score 1 might be linked to factors such as concurrent diseases or comorbidities rather than PE per se. This explanation is underpinned by the fact that increased mortality in MBPEC score 1 was not confirmed in the sensitivity analysis of patients without comorbidities. Therefore, our findings indicate that peripheral LE should be considered as clinically relevant even when treated with anticoagulation.

The prognosis of PE depends on age at diagnosis, sex, signs of right ventricular failure or overload hemodynamic instability, and comorbidity such as COPD or cancer.^12,32,33^ A previous study reported favorable outcome in patients with Charlson comorbidity index score of 0.^33^ In a clinical context it would be of interest to identify patients with increased risk of adverse outcome in this low-risk group. We therefore performed a sensitivity analysis in patients without comorbidities. However, we found no association between mortality and MBPEC/most proximal extension of PE in this population. This strengthens the interpretation that neither MBPEC score nor the most proximal extension of PE should be given decisive clinical importance.

Current guidelines recommend risk stratification based on PESI score.^8^ We therefore performed sensitivity analysis in which we stratified MBPEC according to PESI class I–II and III–V, respectively. However, no clear association with mortality in either group was observed.

We found that patients with MBPEC score 4 more frequently received systemic thrombolysis and were admitted to the intensive care unit more often than those with lower scores. These findings were confirmed in patients without comorbidities. In contrast, we found no association between MBPEC score and mortality. A possible explanation may be that clot burden/distribution per se is often perceived as serious and therefore clinician tend to treat proximal clots more aggressively even in the absence of signs of hemodynamic instability or right ventricular dysfunction. In accordance with current guidelines, our findings regarding the lack of association between MBPEC score 4 and increased mortality do not support such clinical assessments.^13^ However, it must be emphasized that our results should be interpreted with caution as we were unable to adjust for any differences in treatment. Therefore, we cannot exclude that administration of thrombolysis and the more intensive monitoring in patients with MBPEC score 4 may have altered the natural course of the disease, thus impacting our findings.

Although our results indicate that the extent of PE should not be given decisive clinical importance in the risk stratification following PE, we have previously shown an association between Troponin elevation in acute PE and MPBEC score.^24,25^ This indicates that MBPEC might be relevant in contexts other than mortality. Furthermore, there may be clot parameters other than total clot volume/burden that may be important to assess, such as semi-quantitative clot burden measurement, clot volume quantification and lung perfusion quantification using iodine maps, signs of chronicity, as those accurately predict chronic thromboembolic pulmonary hypertension (CTEPH) and other post-PE sequelae.^34,35^

This study has several potential limitations. It was a single center registry-based retrospective study including only patients diagnosed with PE. Our sample size was limited and the morality rate was low, especially in MBPEC 3. Therefore, our results have to be interpreted by caution. Furthermore, patients with incidentally diagnosed PE were included, which might have increased the proportion of patients with comorbidities. On the other hand, the complete, population based inclusion in a well-defined geographic area increases generalizability

A relatively large number of patients were excluded. However, in most cases the exclusions were due to active malignancy. Although a significant proportion of the PE population consists of cancer patients, we considered it relevant to exclude patients with active cancer to improve the assessment of the potential cause of death.

The CTPA reassessments and MBPEC calculations were performed by a single radiologist. However, a previous study has demonstrated excellent MBPEC score inter-rater agreement.^25^ Qanadli or Mastora scores were not assessed, hindering a comparison between MBPEC and a reference standard score. However, excellent correlation between the Qanadli score and MBPEC score has previously been demonstrated.^24^ Finally, type and length of anticoagulation were not available and were thus not adjusted for.

In conclusion, we found no clear association between clot burden as assessed by MBPEC and short-term mortality. However, our results indicate that peripheral PE does not entail a lower risk than centrally located PE.
